# Effects of enrichment removal on cognitive judgement bias of mice: a comparison of two paradigms

**DOI:** 10.3389/fvets.2026.1813283

**Published:** 2026-04-30

**Authors:** Viktoria Siewert, Louisa Bierbaum, Melanie Gleske, Carolin Mundinger, Rupert Palme, Sylvia Kaiser, Norbert Sachser, S. Helene Richter

**Affiliations:** 1 Department of Behavioural Biology, University of Münster, Münster, Germany; 2 Department of Biomedical Science, University of Veterinary Medicine, Vienna, Austria

**Keywords:** affect manipulation, animal emotion, enrichment removal, mice, spatial task, touchscreen task

## Abstract

Cognitive judgement bias (CJB) tests have become indispensable for assessing animal emotions. However, the methodology can vary profoundly. In mice, for example, approaches based on vision, spatial orientation and olfaction have all been used. Yet, the impact of such differences for test outcomes remains largely unexplored. Therefore, we aimed to assess the role of methodological variations for CJB test results by applying two different paradigms for mice: one using visual cues displayed on a touchscreen and another using spatial cues in form of different tunnel lengths. Mice were trained in either paradigm. Upon successful training, the enrichment was removed from their cages to induce differences in affective state. Thereafter, animals were tested for their CJB. Anxiety-like behaviour and faecal corticosterone metabolites (FCMs) served as additional indicators of affective state. Against our expectations, we did not detect effects of enrichment removal on CJB in any paradigm. Likewise, no effects on anxiety-like behaviour were found. By trend, animals showed increased FCMs after enrichment removal. We discuss potential reasons for these findings, focusing on the possibility that the enrichment removal effect might have been too weak. Overall, the influence of methodological characteristics such as cue modality on CJB test results remains an important focus for future studies.

## Introduction

“How are you?”—A simple question for us humans, yet one of the hardest questions to “ask” a non-human animal (henceforth: animal). Due to their inability to speak, animals often leave us in the dark about the emotional states they experience—let alone, whether they do so consciously or not. This is why the assessment of animal affect has become a key challenge across various disciplines, including for example comparative psychology, neuroscience, or animal welfare science.

Over the last two decades, cognitive judgement bias (CJB) tests have become an indispensable tool for emotion assessment in animals (1). They rely on an animal’s interpretation of ambiguous stimuli, with “optimistic” interpretations indicating the anticipation of rewarding outcomes, and “pessimistic” interpretations indicating the anticipation of less rewarding/aversive outcomes (2). CJB paradigms exist for a wide range of animal species (3), but they are all based on the same principle: In a first step, animals are trained to distinguish between two cues (“positive”/CS + and “negative”/CS-), e.g., two different tones. Each cue requires a specific behavioural response. For example, when hearing tone A, animals learn to press a left-sided lever to receive a food reward, whereas when hearing tone B, they learn to press a right-sided lever to avoid a punishment. In a second step, an intermediate tone is played. Pressing the left-sided lever in response to this ambiguous cue would be classified an “optimistic,” and pressing the right-sided lever a “pessimistic” response (2, 4). Recent meta-analyses confirm that both pharmacological as well as non-pharmacological affect manipulations indeed lead to shifts of CJB into the expected directions: Overall, animals in a relatively better state interpret ambiguous stimuli more “optimistically” than animals in a comparably negative state, underlining the validity of CJB tests for the assessment of animal affect (1, 5).

Apart from sharing their basic principle, the CJB tasks described in the literature vary considerably (1, 5), since they have been adapted to species-specific requirements including sensory abilities, food preferences, or simply body size. However, even for animals of the same species, CJB paradigms can vary profoundly, particularly regarding the cue modality they rely on. For example, there are different CJB paradigms for rats that either use auditory, spatial, olfactory, or tactile cues as discriminatory and ambiguous stimuli (6–9). Similarly, there are also spatial, olfactory, tactile, or visual CJB paradigms for mice (10–13). Moreover, the type of reinforcement used can vary strikingly, with some paradigms relying on rewards only (9) while others involve punishments like white noise or foot shocks (6, 14). Also, the involvement of the experimenter can vary, with some tasks being automated, while others require active interventions by the experimenter (13). The methodology of CJB has been discussed frequently (3, 4). So far, however, empirical investigations of such methodological details with regard to test outcomes are still pending. Therefore, we here address the question whether different paradigms for the same species are equally capable of detecting affect-induced differences in CJB in a systematic approach.

We previously developed two different CJB tests for laboratory mice: The “Touchscreen (TS) task” and the “Tunnel (TUN) task.” The TS task is an automated, touchscreen-based paradigm relying on visual cues in form of symbols displayed on a touch-sensitive screen (15). Touchscreen tasks for rodents are particularly valuable for biomedical research questions, since they have the potential of being easily translated to humans (15, 16). Moreover, they provide many automation-related advantages, such as a reduced experimenter effect and the possibility to test several individuals simultaneously (16). The TUN task is based on spatial orientation, using tunnels of different lengths as a cue modality (13). This task is considered to be more ecologically relevant for mice, since spatial navigation through tunnels is a key ability of mice living in complex burrow systems in nature (13, 17).

So far, both tasks were found to meet basic validity criteria: Mice successfully learned both tasks, i.e., they reliably differentiated between “positive” and “negative” training cues. Furthermore, they interpreted the ambiguous cues with reference to the trained cues (13). However, a comparison of both tasks regarding their potential to detect affect-related changes in CJB is still pending. Therefore, in the present study, we ran the TS and the TUN task in parallel to study the effects of the same affect manipulation on the CJB of mice. As an affect manipulation we chose the removal of enrichment from the animals’ home cages, since enrichment loss has already been linked to negative affect in rodents (18–20). In line with this, enrichment removal has been found to induce a “pessimistic” CJB, for example in starlings (21) and pigs (22). Thus, we hypothesised enrichment removal to also exert effects on the CJB of mice in the present study. To verify potential effects of this treatment on complementing measures of affective state, we additionally assessed anxiety-like behaviour in the Elevated plus maze test (EPM) and faecal corticosterone metabolite (FCM) concentrations reflecting hypothalamic-pituitary-adrenal (HPA) axis activity. As for the CJB, we hypothesised enrichment removal to affect the obtained measures.

## Animals and methods

### Animals

The experiment was conducted with a total number of 96 female C57BL/6 J mice that were clinically healthy, free of injury or disease and naïve to behavioural testing. The animals were obtained from Charles River Laboratories (Sulzfeld, Germany) at the age of 8 weeks. Upon arrival, mice were pair-housed in Makrolon cages type III (38 × 23 × 15 cm^3^), containing wood shavings as bedding material (Tierwohl, J. Rettenmaier & Söhne GmbH & Co. KG, Rosenberg, Germany). As enrichment, a paper towel, a cotton wool nestlet, a wooden stick, a white cardboard house (height: 6 cm, bottom area: 117 cm^2^) and a semi-transparent red plastic tunnel hanging from the cage lid (diameter: 5.5 cm, length; 10 cm, Datesand Group, Bredbury, United Kingdom) were provided. Mice were housed under a reversed 12-h dark/light cycle with lights off at 9:00 a.m., a temperature of about 22 °C and a relative humidity of about 50%. Before the start of the experiment, mice were provided with water and food (Altromin 1,324, Altromin Spezialfutter GmbH & Co. KG, Lage, Germany) *ad libitum*. Starting with the CJB training phase (for details see *Experimental Design*), all animals received a mild food restriction that is commonly applied in order to increase training motivation during CJB tasks (13, 23, 24). Therefore, each cage was provided with a predetermined amount of food per day, tailored to the animals’ body weights to maintain them at 90–95% of individual *ad libitum* feeding weights.

### Experimental design

**Figure 1:**
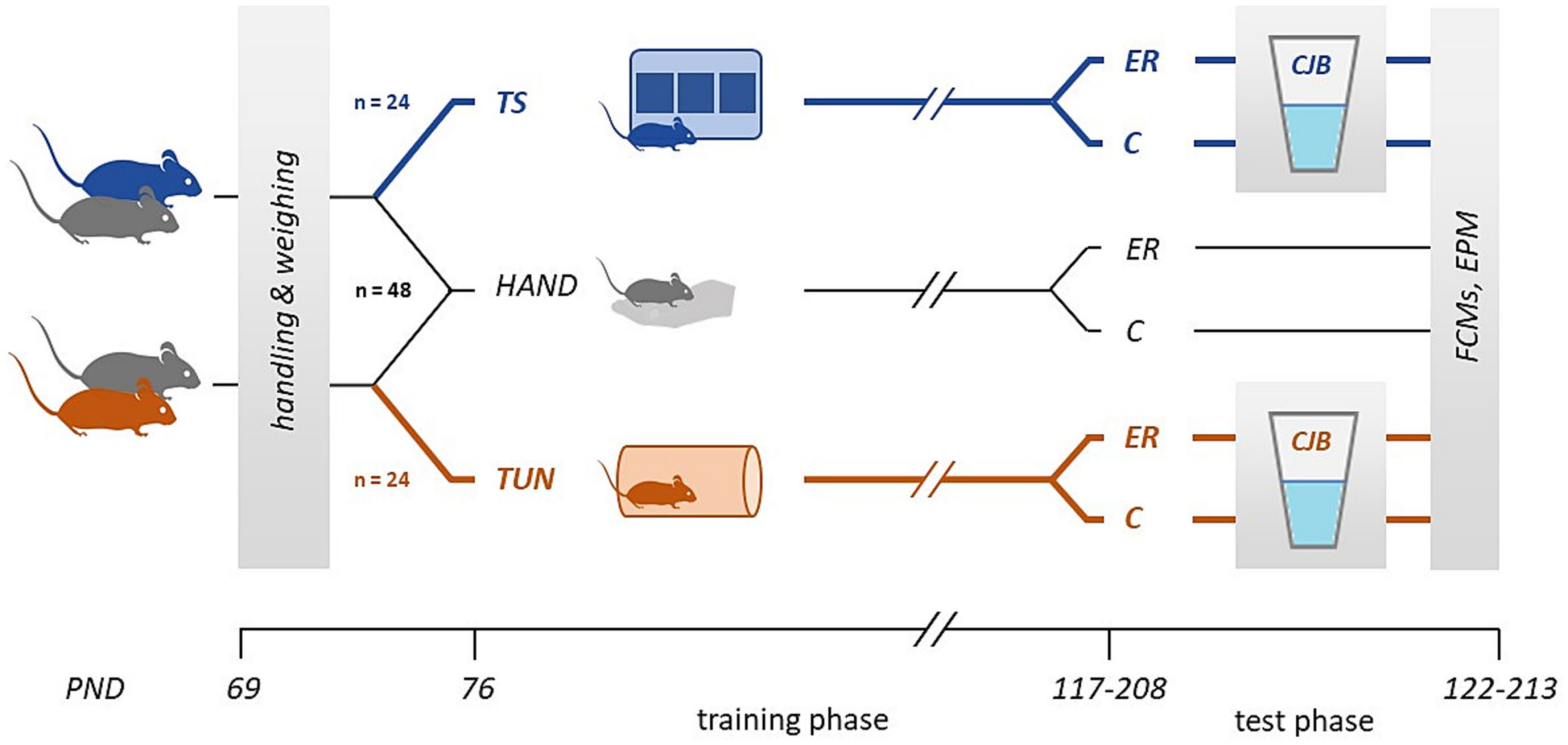
Experimental design. After a week of handling and daily weighing, the training phase started. Mice were assigned to one of three groups: the touchscreen (TS), tunnel (TUN), or handling (HAND) group. Per cage, always one mouse was trained in either task, while the other individual belonged to the HAND group. Once a mouse finished TS/TUN training, the respective individual and its cage mate received an affect manipulation (ER, enrichment removal; C, control). Trained mice were subsequently tested for their cognitive judgement bias (CJB). Faecal corticosterone metabolites (FCMs) were extracted from faeces produced during the time of CJB testing by all groups of mice. At the end of the test phase, all animals were tested in the elevated plus maze (EPM). TS/TUN mice could be excluded from the study due to learning difficulties, which in some cases also required the exclusion of cage mates, leading to the different sample sizes during the test phase (for details on exclusion criteria see text).

The study was split into six cohorts of mice that were tested successively by two experimenters [3 cohorts per experimenter, in the following also referred to as “batches”; see (25) for advantages of heterogenizing study samples this way]. The experimental design is depicted in Figure 1. From postnatal day (PND) 69, all mice were weighed and handled on a daily basis using the cup-handling technique (26). At PND 76, the training phase started. From then on, 24 mice were trained in the TS task (“TS group”), and 24 mice in the TUN task (“TUN group”). Training durations may vary considerably between individuals and tasks (13). Therefore, we did not house TS and TUN mice within the same cage, but together with a companion mouse each. These companion mice constituted the third experimental group that was only weighed and handled daily (“HAND group,” *n* = 48). After successful training of each TS/TUN mouse, the test phase for this individual and its cage companion started. At the beginning of the test phase, half of the cages experienced an affect manipulation in form of enrichment removal (“ER”). More specifically, all enrichment items except the red plastic tunnel were removed from the home cages. The tunnel was left in the cages for welfare reasons. The other half of mice served as a control, with the enrichment remaining the same as before (control; “C”). Afterwards, the CJB test was carried out across five consecutive days. The faeces dropped by all mice during the time of testing were collected for FCM analysis. Subsequently, an Elevated plus maze test (EPM) was conducted to assess anxiety-like behaviour.

### The touchscreen task

#### Apparatus

**Figure 2:**
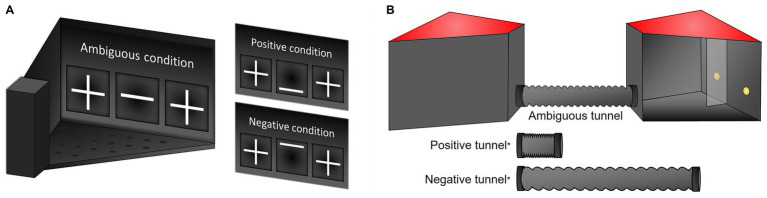
Apparatuses for cognitive judgement bias testing. **(A)** TS task: Touchscreen chamber with “touch fields” displaying crosses as touch symbols (left and right) and a central “cue presentation” field, here displaying the ambiguous middle cue. **(B)** TUN task: Tunnel apparatus with two chambers, connected via a tunnel (here: intermediate tunnel, 31 cm). Left chamber: View from the outside, right chamber: view from the inside. In each chamber, the rear wall contained two reward holes which could be illuminated by LED lights. The holes were separated by a small Perspex wall located in the middle of the rear wall.

For the TS task, an automated touchscreen system for mice was used [Bussey-Saksida Mouse Touch Screen Chambers, Model 80,614, Campden Instruments Ltd., Loughborough, Leics., United Kingdom; for a detailed description of the apparatus see (13)]. The system consisted of four independent, trapezoid-shaped chambers. Each chamber was equipped with a touchscreen at the front and a reward dispenser at the rear end. As rewards, servings of sweet condensed milk were used (Nestlé “Milchmädchen gezuckerte Kondensmilch”; diluted 1:4 in tap water; in the following: “SCM”). The touchscreen was covered by a black Perspex mask, allowing access to the screen via three windows: a left and right “touch field” and a “cue presentation field” in the middle (Figure 2A). Data from TS training and testing was recorded automatically using the software ABET II (version 2.0, Campden Instruments Ltd., Loughborough, Leics.).

#### Paradigm

The training was split into several training steps with increasing complexity that have been described in detail previously (13, 23, 27, 28). Briefly, mice were trained to distinguish between bars displayed on the central “cue presentation field” as follows: When a bar was displayed on the lower end of the central window (= “positive cue”), mice had to touch a cross on the left “touch field” to receive a large reward (12 μL of SCM). Touching the cross on the right “touch field” resulted in a small reward only (4 μL SCM). When a bar was displayed on the upper end of the central window (= “negative cue”), mice had to touch the right cross to receive a small reward (4 μL SCM). Touching the left cross resulted in a mild punishment, i.e., 10 s during which no next trial could be initiated, alongside an illumination of the touchscreen chamber. The correct touch field in response to positive and negative cues was counterbalanced across the animals (for details regarding the specific training steps and learning criteria, see Supplementary material).

As soon as mice successfully learned this discrimination task, they proceeded to the CJB test. The test was conducted across five sessions (Monday–Friday). During each test session, mice were presented with 44 regular training trials and 6 additional, ambiguous probe trials that were pseudo-randomly interspersed between training trials. As ambiguous cues, bars displayed at three intermediate positions on the “cue presentation field” were used: “near positive” (NP; 4 cm below upper edge), “middle” (M; 3 cm below upper edge), and “near negative” (NN; 2 cm below upper edge). Responses to the ambiguous cues were left un-rewarded/un-punished. Across the test phase, a total number of 220 training (110 x P, 110 x N) and 30 test trials (10 x NP, 10 x M, 10 x NN) was presented.

#### Procedure

Training and testing were carried out on a daily basis (Monday–Friday) with one training session per day and mouse, scheduled between 12:00–14:00 p.m. For each session, a mouse was taken out of its home cage, weighed, and transported to the adjacent room within a semi-transparent, red plastic box (21 × 21 × 15 cm^3^). It was then placed into a touchscreen chamber and the training/test session was started. On each training day, each mouse encountered the training step that matched its individual training level. Sessions lasted maximally 30 min or less if an animal met the training step-specific criterion in a shorter time. After each session, mice were transported back to their home cages. The order of mice that were trained on the same day was randomised. The touchscreen chambers were wiped with water between mice. As soon as the daily training/testing was finished for all animals, cages were fed their daily food ration.

#### Exclusion criteria

Mice could be excluded from the study if they did not learn the task within a predetermined time window. More specifically, if after 90 days of training mice had not reached the final training step, they were excluded (for details regarding training steps, see Supplementary material). In such a case, their cages were provided with *ad libitum* diet again (i.e., also cage mates of the HAND group received *ad libitum* food again). Due to a potential influence of this change in feeding regime, both animals of the cage were also excluded from the EPM and FCM analysis. Furthermore, if the mice successfully completed training but did not maintain a response accuracy of at least 80% towards both the positive and negative cue during the CJB test phase, their CJB test results were not included in the analysis.

### The tunnel task

#### Apparatus

For the TUN task, a modified version of the apparatus described previously was used (13). It consisted of two trapezoid-shaped, grey Perspex chambers (base area ca. 400 cm^2^, height: 24.5 cm), each allowing entrance to a connection tunnel via a hole in the front side (∅ 5 cm) that could be closed by a sliding door (Figure 2B). The tunnel consisted of a 50 cm long, transparent PVC extraction hose and could be compressed to shorter lengths (40.5 cm, 31 cm, 21.5 cm, and 12 cm). The tunnel was placed into a length-matching guide rail to prevent bending. Chambers were closed by semi-transparent, red plastic lids during training and testing. Two reward holes (“left” and “right”; ∅ 1.5 cm) were located in the rear wall of each chamber. They could be closed by small sliding doors. Once opened, the holes were illuminated by LEDs positioned outside of the chamber. The reward holes were spatially separated by a dividing wall midway between them (width: 6.5 cm). The two chambers were used as “start” and “goal” in an alternating manner: Mice were placed into one of the chambers at the beginning of a session and had to traverse the tunnel to reach the opposite chamber, where they could receive a reward. Upon completion of one such trial, the direction was reversed, i.e., mice had to traverse the tunnel in the opposite direction, and could receive a reward in the opposite chamber. As rewards, servings of SCM were used and provided by the experimenter via a pipette inserted into the holes from behind the chamber. Data from TUN training and testing were recorded manually.

#### Paradigm

As for the TS task, TUN training was split into several training steps that increased in complexity (for details see (13); Supplementary material). The animals learned to distinguish between tunnels of two different lengths: “short” (12 cm), and “long” (50 cm). The two tunnel lengths constituted either the “positive” (P) or “negative” (N) cue. When traversing the “positive” tunnel, mice could receive a large reward (12 μL SCM) when nose poking into one of the reward holes (left or right). When traversing the “negative” tunnel, they had to nose poke into the respective other hole to receive only a small reward (4 μL SCM). Upon nose pokes into the incorrect reward hole, no reward was delivered and the reward hole was closed immediately. The association of tunnel length and correct reward hole remained the same for each individual but was counterbalanced across animals.

As soon as mice had successfully learned the task, they proceeded to the CJB test. Again, the test was conducted across five sessions (Monday–Friday). During each session, mice encountered 30 regular training trials and 3 additional, ambiguous probe trials that were pseudo-randomly interspersed between the training trials. As ambiguous cues, tunnels of the three intermediate lengths (21.5 cm, 31 cm, and 40.5 cm) were used, classified as “near positive” (NP), “middle” (M) and “near negative” (NN). In contrast to the TS task, in the TUN task, responses to the ambiguous cues were rewarded according to the animals’ expectations: If choosing the reward hole associated with the large reward, they received 12 μL SCM, if choosing the reward hole associated with the small reward, they received 4 μL of SCM. Across the test phase, a total number of 150 training (75 × P, 75 × N) and 15 test trials (5 × NP, 5 × M, 5 × NN) was presented.

#### Procedure

Training and testing were carried out on a daily basis (Monday–Friday) with one training session per day and mouse, scheduled between 9:00–12:00 a.m. and took place in the housing room of the mice under red light. For each session, a mouse was taken out of its home cage, weighed, and placed into the apparatus, before the session was started. On each training day, each mouse encountered the training step that matched its individual training level. Sessions lasted maximally 30 min or less if an animal met the training step-specific criterion in a shorter time. After each session, mice were placed back into their home cages. The order of mice that were trained on the same day was randomised. Between mice, the apparatus was wiped with 70% ethanol and a clean tunnel was installed. As soon as the daily training was finished for all animals, cages were provided with a pre-determined amount of food.

#### Exclusion criteria

Also TUN mice could be excluded from the study if they did not learn the task within a predetermined time window. Mice are usually able to perform the first training step within 5 sessions. We here set the limit to a maximum of 5 additional training sessions, after which mice were excluded from the study if they still had not met the criterion (for details on training steps, see Supplementary material). As for the TS task, this also meant the simultaneous exclusion of the handled cage mate. Moreover, if the animals’ performance during the CJB test dropped below 80% correct response towards the training cues, their optimism scores were excluded from the analysis.

### Elevated plus maze test

The EPM is commonly used to assess anxiety-like behaviour in mice (29–31). The apparatus consisted of a plus-shaped maze consisting of grey PVC and elevated 50 cm above the floor, with two open and two closed arms (length: 30 cm, width: 5 cm) extending from a central square (5 cm^2^). The open arms were surrounded by a 0.5 cm high border to prevent mice from falling down. The closed arms were surrounded by 20 cm high walls. An LED lamp with an illuminance of approximately 25 lux was installed centrally above the maze. Behaviour of the mice was videorecorded (camera: DMK 22AUC03, The Imaging Source, Bremen, DE) and automatically tracked using a tracking software (ANY-maze Video Tracking System, version 6.32, Stoelting Co., Wood Dale, United States).

Mice were tested exactly 3 h after their last CJB test session, or, in case they belonged to the HAND group that did not undergo CJB testing, immediately after their cage mates. Before each test, the apparatus was cleaned with 70% ethanol. Mice were transported from their housing room to the testing room within an empty Makrolon Type II cage (23 × 17 × 14 cm), shielded from daylight using a black cloth. Upon arrival in the testing room, mice spent 1 min in the transport cage to acclimatise before testing. They were then placed in the central area of the EPM, always facing the same closed arm. The experimenter left the room and mice could freely explore the apparatus for 5 min. Parameters analysed were the relative time the mice spent on the open arms of the EPM, the entries made into the open arms and the total distance travelled on the apparatus.

### Faecal corticosterone metabolites

Basal adrenocortical activity of the animals was monitored non-invasively by analysing corticosterone metabolites from the faeces the mice produced during the time of CJB testing. During the dark phase, a peak of FCMs in response to a stressor can be found 4–6 h after exposure (32). By collecting faeces during the time of CJB testing, any short-term influence of the test itself was ruled out. When mice were tested for their CJB in either the TS or the TUN task, all faeces left in the respective apparatus during the five test sessions were collected. Furthermore, faeces from the respective cage companions of the HAND group were sampled during the exact same time span. For this, HAND mice were transferred to a waiting cage, i.e., a novel clean cage containing a thin layer of bedding material and the home cage enrichment (if present), and all faeces produced in this cage were collected.

Samples were frozen at −20 °C after collection. Subsequently, they were dried, homogenised and aliquots of 0.05 g were extracted with 1 mL of 80% methanol. If a sample contained less than 0.05 g of material, the amount of methanol was adjusted. Samples were then analysed using a 5α-pregnane-3β,11β,21-triol-20-one enzyme immunoassay (32, 33).

### Statistics

All statistical analyses were conducted in R (34) using the packages “lme4” (35) and “lmerTest” (36) for mixed model fitting. All plots were created using the “ggplot2” package (37).

#### Analysis of cognitive judgement bias training

A total number of 22 TS and 21 TUN mice successfully completed training and CJB testing. According to our predetermined exclusion criteria, one mouse had to be excluded from TS and one mouse from TUN training before testing. In addition, two TUN mice did not maintain the learning criterion of 80% correct responses towards the training cues during the CJB test phase, so their CJB data was excluded from the analysis. Furthermore, another TS-trained mouse was excluded, however, not according to our *a priori* criterion, but due to the fact that it encountered unforeseen, severe learning difficulties already at an early training stage. Specifically, the mouse had to return to the basic discrimination training step multiple times during the course of training, due to recurrent declines in performance (for details on the so-called “return criterion,” see Supplementary material). Since no consistent improvement in performance was observed after four return cycles and more than 70 training sessions, we concluded that the animal was unlikely to accomplish training within a reasonable time window.

Not only the number of excluded animals but also the training duration is methodologically relevant. Extensive training may influence the animals’ affective state, which could in turn affect CJB test outcomes. Additionally, training duration is relevant for the practical implementation and feasibility of the paradigms (4). We therefore compared the training durations of mice between the TS and TUN task by fitting a Linear Mixed Model (LMM) including the task the animals underwent (with the two levels TS and TUN) and the experimenter who trained them (two levels: experimenter 1, experimenter 2) as fixed factors. “Batch” (levels: 1–6) was included as a random factor.


Total number of training days~task+experimenter+(1∣batch)

Residuals were examined for heteroscedasticity and normal distribution using the DHARMa package (38). To meet the assumptions for the applied analysis, the data was log-transformed.

#### Analysis of cognitive CJB test

During the CJB test, mice could respond towards the cues as if predicting a positive or negative outcome, respectively. Responses according to the P cue were classified as “optimistic,” responses according to the N cue as “pessimistic” (note that this was also the case for both reference cues, i.e., an “optimistic” response to the P and a “pessimistic” response to the N cue simply equal the respective “correct” response). To illustrate the animals’ optimism levels graphically, we calculated an “optimism score” for each mouse and each cue according to the following formula:


$$\text{Optimism score}=\frac{\begin{array}{l} N\;\text{choices}\;{(}^{"}{\text{optimistic}}^{"})\\ -N\;\text{choices}\;{(}^{"}{\text{pessimistic}}^{"}) \end{array}}{N\;\text{choices}\;(\begin{array}{l} "{\text{optimistic}}^{"}\\ {+}^{"}{\text{pessimistic}}^{"} \end{array})}$$

Scores could range from +1 to −1, with higher scores indicating a higher proportion of “optimistic” choices. For the analysis of the CJB data, we fitted General Linear Mixed Models (GLMMs), modelling the absolute number of optimistic versus pessimistic choices for both the TS and TUN task. To estimate the influence of the affect manipulation (with two levels: ER, C) and cue type (with five levels: P, NP, M, NN, N), these variables, as well as their interaction, were included as fixed factors in our model. Additionally, the experimenter was included as a fixed factor (with two levels: experimenter 1, experimenter 2) and “ID” (describing the individual) as a random factor.

We then explored models including “age” (reflecting the age at the time of CJB testing and, at the same time, training duration) and “batch” (the cohort the animals were tested in) as random effects, aiming to identify the optimal random effects structure. Comparing the Akaike information criterion (AIC) across models (39), we did not find support for “age” or “batch” to add improvement to the model fit (for statistical details, see Supplementary material). On this basis, we retained “ID” as a relevant random effect, leading to the following, most parsimonious model:


$$\begin{array}{l} \text{Choice}\sim {\text{Affect manipulation}}^{*}\;\mathrm{Cue}+\text{Experimenter}\\ +(1|\mathrm{ID}),\text{family}=\text{binomial}) \end{array}$$

Model residuals were examined for heteroscedasticity and normal distribution using the DHARMa package (38). Pairwise post-hoc comparisons of responses to the different cues were conducted using the “emmeans” package (40) (adjust = “tukey”).

#### Elevated plus maze

A total number of 89 mice (45 HAND mice, 22 TS mice and 22 TUN) mice were tested in the EPM. Six animals were excluded from EPM testing in line with the aforementioned exclusion criteria (three mice that did not complete training plus their three cage partners). Moreover, data of one mouse were erroneously not recorded and could thus not be included in the analysis, amounting to a total number of 7 exclusions.

Outcome variables were fitted using LMMs. To estimate the effect of the affect manipulation on trained as well as handled mice, we included “affect manipulation” (with two levels: ER, C) and “group” (with three levels: TS, TUN, HAND), as well as their interaction as fixed factors in our model. Additionally, the experimenter was included as a fixed factor (with two levels: experimenter 1, experimenter 2).

As for the CJB data, we again aimed to identify the optimal random effects structure by exploring models including “age” (reflecting the age at the time of CJB testing and, at the same time, training duration), “batch” (the cohort the animals were tested in), and “cage” (the home cage shared by always one TS or TUN and one HAND mouse) as random effects. Comparing the AIC across models (39), we retained “cage” as a relevant random effect, leading to the following, most parsimonious model:


$$\text{Behaviour}\sim {\text{affect manipulation}}^{*}\;\text{group}+\text{experimenter}+(1|\text{cage})$$

Model residuals were examined for heteroscedasticity and normal distribution using the DHARMa package (38). Pairwise post-hoc comparisons between the different groups (TS, TUN and HAND) were conducted using the “emmeans” package (40) (adjust = “tukey”).

#### Faecal corticosterone metabolites

We obtained a sufficient amount of faeces for the analysis from only 59 individuals (n_HAND-C_ = 21, n_HAND-ER_ = 21, n_TS-C_ = 5, n_TS-ER_ = 6; n_TUN-C_ = 3, n_TUN-ER_ = 3). This was mainly due to the fact that animals generally defecated very little during CJB testing. Furthermore, the sampling duration in the present study was slightly shorter than in other studies [3 h are common (41)]; we here sampled during testing/waiting, which resulted in a mean sampling duration of about 70 min per individual).

In light of the particularly low sample sizes for the TS and TUN group, we pooled the data across the three groups of mice (HAND, TS, TUN) and analysed the effect of the enrichment removal on the animals’ FCMs using a linear model (LM):


FCMs~affect manipulation

Model residuals were examined for heteroscedasticity and normal distribution using the DHARMa package (38).

## Results

### Training

**Figure 3:**
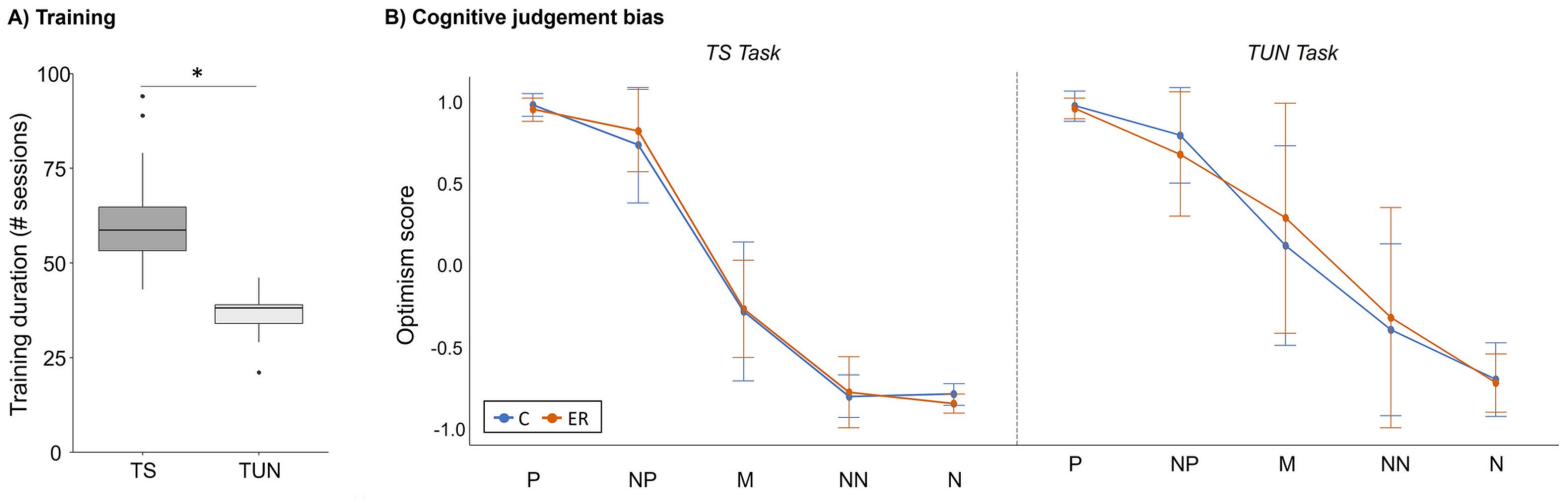
Cognitive judgement bias (CJB) training and test. **(A)** Training durations. Data are shown as medians (horizontal lines) with inter-quartile ranges (boxes), 1.5 x inter-quartile range (whiskers) and outliers (dots). TS: Touchscreen task, TUN: Tunnel Task. Statistics: LMM revealed a significant difference in training durations between TS and TUN mice, *p < 0.001. Sample sizes: *n*_TS_ = 22, *n*_TS_ = 21. **(B)** Cognitive judgement bias (CJB) of mice in the TS and TUN task. After completion of training, the enrichment (except for the red plastic tunnel) was removed from the home cages of half of the mice (“ER”), while it remained in the home cages of the other half (“C”). Data are depicted as optimism scores (means ± SD) for all five cues (P, positive; NP, near positive; M, middle; NN, near negative; N, negative). Sample sizes: *n*_TS-C_ = 10, *n*_TS-ER_ = 12, *n*_TU-C_ = 11, *n*_TU-ER_ = 10.

The analysis of training durations revealed that TS mice (i.e., mice trained in the touchscreen paradigm) needed significantly longer to complete training than TUN mice (i.e., mice trained in the tunnel paradigm; LMM: *F*_(1,36.24)_ = 83.35; *p* < 0.001; Figure 3A). We did not detect an effect of the experimenter on training durations (LMM: *F*_(1,4.01)_ = 0.52; *p* = 0.63).

### Cognitive judgement bias

The analysis of the CJB test revealed a significant effect of “cue” (5 levels: P = positive, NP = near positive, M = middle, NN = near negative, N = negative) for both the TS (GLMM: χ^2^ = 5.1, df = 4, *p* < 0.001; Figure 3B) and TUN task (GLMM: χ^2^ = 591.97, df = 4, *p* < 0.001; Figure 3B). For the TS task, subsequent pairwise comparisons showed that mice interpreted four of the five adjoining cues significantly differently (*p* < 0.001 for the following comparisons: P-NP; NP-M, M-NN; for statistical details, see Supplementary material). Only between the NN vs. N cue, no significant difference in optimism scores was detected (*p* = 0.94). In the TUN task, mice interpreted all five adjoining cues significantly differently (*p* < 0.01; for statistical details, see Supplementary material).

Regarding the enrichment removal, the analysis revealed no significant effect of “affect manipulation” nor a significant interaction between “affect manipulation” and “cue” on the animals’ optimism scores, neither for the TS task (GLMM: χ^2^_affect manipulation_ = 0.08, df = 1, *p* = 0.78; GLMM: χ^2^_affect manipulation x cue_ = 5.91, df = 4, *p* = 0.21), nor for the TUN task (GLMM: χ^2^_affect manipulation_ = 0.52, df = 1, *p* = 0.47; GLMM: χ^2^_affect manipulation x cue_ = 1.62, df = 4, *p* = 0.81). By trend, there was an effect of the experimenter on CJB in the TS task (GLMM: χ^2^ = 3.59, df = 1, *p* = 0.06), but not in the TUN task (GLMM: χ^2^ = 0.14, df = 1, *p* = 0.71).

### Anxiety-like behaviour and faecal corticosterone metabolites

**Figure 4:**
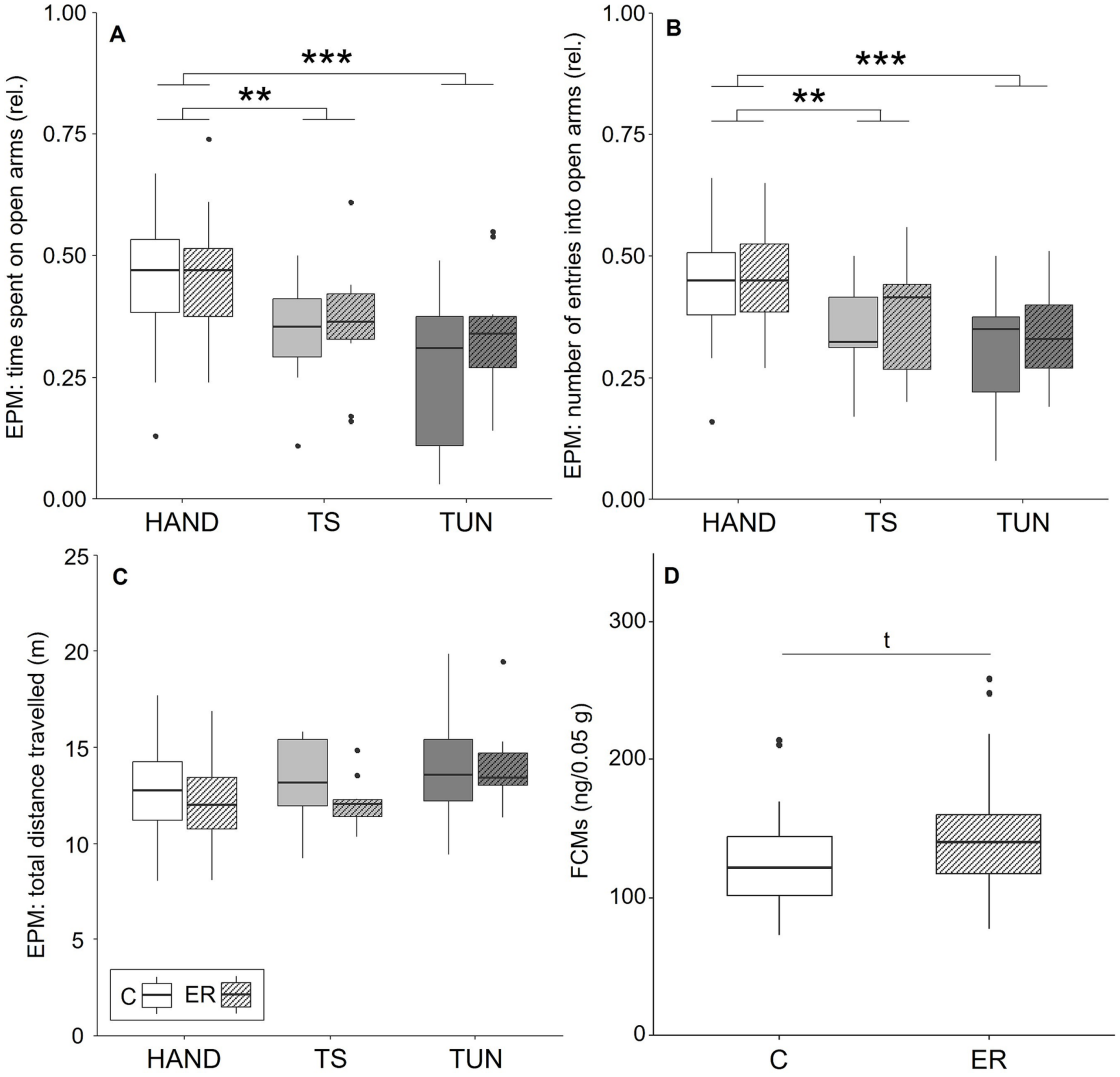
Results of the elevated plus maze (EPM) and faecal corticosterone metabolites (FCMs). **(A)** Relative time spent on and **(B)** relative number of entries made into the open arms of the EPM. **(C)** Total distance travelled on the EPM. **(D)** FCMs. Data are shown as medians (horizontal lines) with inter-quartile ranges (boxes), 1.5 x inter-quartile range (whiskers) and outliers (dots). Experimental groups: HAND = handled control group, TS = touchscreen-trained and -tested group, TUN = tunnel-trained and -tested group; Affect manipulations: C = control, ER = enrichment removal. Sample sizes **(A–C)**: *n*HAND-C = 22, *n*HAND-ER = 23, *n*TS-C = 10, *n*TS-ER = 12; *n*TUN-C = 11, *n*TUN-ER = 11. Sample sizes (D): *n*C = 29, *n*ER = 30. tp < 0.1, **p < 0.01, ***p < 0.001.

The analysis of the animals’ behaviour on the EPM did not reveal any significant effects of the “affect manipulation,” i.e., enrichment removal, on any of the parameters obtained (LMMs for: time the mice spent on the open arms: χ^2^ < 0.001, df = 1, *p* = 0.99, Figure 4A; relative number of entries they made into the open arms: χ^2^ < 0.001, df = 1, *p* = 0.98, Figure 4B; total distance the animals travelled on the apparatus: χ^2^ = 0.59, df = 1, *p* = 0.44 Figure 4C). There was also no significant interaction between the affect manipulation and the group the mice belonged to (TS, TUN or HAND; for statistical details, see Supplementary material).

However, there was a significant main effect of “group” on the time the mice spent on the open arms (LMM: χ^2^ = 21.89, df = 2, *p* < 0.001) and the number of entries they made into the open arms of the EPM (LMM: χ^2^ = 20.19, df = 2, *p* < 0.001). *Post-hoc* comparisons revealed that both trained groups, i.e., TS and TUN mice, spent significantly less time on the open arms (TS-HAND: *p* < 0.01; TUN-HAND: *p* < 0.001) and entered these significantly less often compared to HAND mice (TS-HAND: *p* < 0.01; TUN-HAND: *p* < 0.001).

Furthermore, the experimenter significantly affected the time the mice spent on the open arms of the EPM (LMM: χ^2^ = 6.94, df = 1, *p* < 0.01) and the number of entries made into the open arms (LMM: LMM: χ^2^ = 7.63, df = 1, *p* < 0.01).

The analysis of FCMs revealed a trend for an effect of the enrichment removal (LM: *F* = 2.95, df = 1, *p* = 0.09), with ER mice displaying higher FCMs compared to C mice (Figure 4D).

## Discussion

CJB tests show considerable variation both between and within species. However, the impact of such methodological differences for test outcomes remains largely unexplored. To address this issue, we conducted two CJB tests in mice to evaluate their potential to detect affect-related changes. Importantly, every CJB paradigm comes with methodological characteristics that can influence outcomes beyond single factors. In our case, for example, one task was based on visual cues, the other on spatial ones. Furthermore, one was automated, whereas the other was conducted manually. Also, the animals completed different numbers of trials in each task, and the rules learned during training differed slightly. This highlights that each established paradigm carries its own methodological distinctiveness. Consequently, any observed differences in test outcomes should be interpreted as differences between two tasks that each represent an independent methodological “package.”

Summarising our findings, the results of both CJB tasks resembled each other, while we did not detect differences in CJB as a consequence of enrichment removal in any of them. Furthermore, no effects of enrichment removal on anxiety-like behaviour were detected, while our data hint at an effect of the affect manipulation on HPA axis activity.

### Both CJB tasks fulfil basic validity criteria

Both the TS and the TUN task were learned successfully by most animals, with higher training durations needed for the TS task. This is consistent with previous results (13). The TUN task is considered to be ecologically more relevant for mice than the TS task, as it relies on tunnels as spatial cues, thereby promoting the natural behaviour of mice to navigate through complex burrow systems (13, 42). Thus, our data support the assumption that the ecological relevance of a task facilitates discrimination learning (13). Additionally, the visual abilities of the tested mice may contribute to differences in learning performance. Although visual impairments are generally considered less common in C57BL/6 J mice than in C57BL/6 N substrains (43), subtle variation in visual function could nevertheless influence learning speed in the TS task. In total, 5 mice were excluded from the CJB test due to learning difficulties (2 TS mice and 3 TUN mice). It is generally aimed to prevent exclusions, since this can bias the overall study sample towards animals possessing specific cognitive abilities (4). We here aimed to minimise a selection bias towards “learners” by training animals over relatively long time spans.

During testing, responses of the mice in both paradigms differed between the five cues and formed a graded response curve that is typical for CJB tests across species (44). This underlines that mice interpreted the ambiguous cues (NP, M, NN) with reference to the previously learned positive and negative cues. Thus, both tasks fulfil the basic validity criteria for CJB paradigms (44, 45).

### Effects of enrichment removal on CJB

Neither in the TS nor in the TUN task, an effect of enrichment removal on CJB could be detected. This was rather unexpected, as effects of enriched housing conditions on CJB have been reported consistently across species (9, 11, 22, 46, 47). In particular, the loss of enrichment has been associated with a negative CJB (21, 22). While the underlying causes of our findings remain difficult to determine, the following methodological aspects might have influenced and/or confounded the results we obtained:

First, the affect manipulation we applied might have been weaker than expected. Reconsidering the procedure, it is important to note that the semi-transparent, red plastic tunnel was left in the cages of the mice in our study during the treatment for welfare reasons. Previous studies on enrichment for mice have shown that even few enrichment items, particularly nesting material, can exert a considerable impact on the animals (48–50). Even though the tunnel is not considered as nesting material, it still serves as a shelter, thereby sharing an important characteristic with nest structures. Therefore, it is possible that a more pronounced effect could have been observed as a result of removing *all* enrichment items from the animals’ cages. Moreover, the previous studies reporting enrichment effects on CJB have provided a broader range and greater complexity of enrichment devices. These included combinations of structural elements such as tunnels, shelters and platforms (9, 11, 47, 48), manipulable objects like wooden or cardboard items (9, 22, 46, 47), as well as additional nesting material or deeper substrates (9, 11, 46, 47). Importantly, some studies also introduced novel items or regularly replaced enrichment objects, thereby increasing environmental variability and novelty (22, 47). In contrast, the comparatively limited and static enrichment in our study may have reduced the potential impact of its removal.

Second, it has been discussed previously that training procedures, as they are commonly applied for CJB tests, may themselves influence the animals’ affective state, and therefore could mask potential treatment effects (4, 24, 41, 51). Indeed, we detected pronounced effects of training in the TS and TUN task on the animals’ anxiety-like behaviour in the EPM as compared to HAND animals (for details see discussion below). This supports the assumption that a potential effect of enrichment removal might have been masked by the daily training sessions the mice underwent.

Third, the failure to detect an effect of enrichment removal might have resulted from the sample size being too small, particularly in light of a potentially weaker effect than expected (due to a possibly ineffective treatment and confounding training effects, see discussion before). The choice of sample sizes in the present study (*n* = 12 per group and paradigm) was guided by previous studies investigating enrichment effects on CJB in rodents [for example: Brydges et al.: *n* = 6 per group (9); Richter et al.: *n* = 6 per group and strain (46)]. Another study by Resasco and colleagues (11) recently confirmed a large effect of structural enrichment on the CJB of mice using an olfactory CJB paradigm with a sample size of *n* = 16 (n_1_ = 7, n_2_ = 9; effect size: *Cohen’s d* = 1.083). However, except for Resasco et al., to the best of our knowledge, no study in mice detected effects of an affect manipulation on CJB (24, 27, 28). Against this background, the following section discusses some general challenges of CJB tasks.

An ongoing matter of discussion are the cognitive processes involved in CJB task performance. More specifically, the interpretation of ambiguous stimuli is assumed to be guided by a range of cognitive processes that are part of decision-making, for example risk taking, how individuals value rewards versus punishers, or how they direct their attention towards environmental cues. The involvement of all these components renders the direct inference of “optimism”/“pessimism” of CJB tasks as indicators of emotional states even more complex (3, 52, 53). Within this context, particularly the effect of using food rewards during training/testing, combined with a caloric restriction, has been discussed as a potential confounding factor and could influence response tendencies (54). Since paradigms based on food rewards and/or diet restriction are still common practice, it appears especially important to disentangle the influence of feeding motivation on CJB in future studies. Moreover, it is still a matter of discussion how animals actually perceive the ambiguous stimuli in a CJB task. On the one hand, the ambiguous cue might (to a certain degree) be perceived as “novel,” instead of solely ambiguous (52). Also, the perceived similarity of the ambiguous cues to the reference cues can affect test outcomes (52). Thus, animals might not generalise the ambiguous cues as expected, but generalisation curves might be skewed and non-linear. In other words: The “middle” of any metric scale we presuppose (e.g., the intermediate bar position on a screen or the intermediate tunnel length) might not be the perceived “middle” for the animals (4, 5).

Overall, both methodological as well as conceptual considerations may aid in explaining the fact that we did not detect an effect of enrichment removal on the CJB of mice in the present study. Future studies might help to disentangle the above discussed influencing factors, pursuing a more comprehensive understanding of the link between CJB and emotional states in animals. In this regard, theoretical ideas to refine the perceived ambiguity of intermediate cues have already been developed (52). Investigating these in empirical studies would constitute a promising next step.

### Effects of enrichment removal on anxiety-like behaviour and FCMs

No effects of enrichment removal on anxiety-like behaviour could be detected. Again, this finding contradicts our expectations, since enrichment provision and removal have been reported to affect anxiety-like behaviour in rodents (55–57). However, this finding is consistent with the results of the CJB tests, where we also did not detect an influence of the applied affect manipulation. As already discussed above in more detail, we retained the semi-transparent, red plastic tunnel in the cages, while all other enrichment items were removed during the treatment. This might have alleviated a potential effect of the procedure, which was thus not detectable in both the CJB tests and the EPM. Thus, the EPM results provide additional support for the possibility that the enrichment removal procedure was not sufficiently effective.

However, by trend, mice that experienced enrichment removal displayed elevated FCM concentrations compared to control animals, indicating an increase in HPA axis activation. In light of the generally negative influence of enrichment removal on affective state observed in previous studies (18–20), the trend we found here might thus indicate a state of negative arousal. This finding appears reasonable in light of the existing literature: Effects of environmental enrichment on HPA axis activity in rodents have been reported previously, even though the direction of the effects was found to depend on further factors, such as strain or compilation of structural enrichment items (18, 49, 58, 59).

Taken together, there might have been a small effect of the affect manipulation that was only detectable at an endocrinological level, but that was not reflected in CJB or anxiety-like behaviour as assessed in this study.

### Effects of group and experimenter on affective state

We included the two factors “group” and “experimenter” in our analysis (for details see material and methods). With respect to the animals’ anxiety-like behaviour, we found pronounced effects of the group the animals belonged to (TS, TUN or HAND). Specifically, mice of the two trained groups, i.e., TS and TUN, displayed higher levels of anxiety-like behaviour compared to mice that were only handled. The finding that training procedures can affect anxiety-like behaviour is consistent with results of previous studies conducted in our lab and has been discussed extensively before. Briefly, the observed effects might be due to an anticipation of reward that was built up during regular training sessions, and which might have been violated by being placed into an EPM apparatus, causing a putative state of negative affect and leading to the increase in anxiety-like behaviour (41, 51). Moreover, also the experimenter affected the animals’ anxiety-like behaviour. It is well known that the experimenter can affect test outcomes of behavioural studies considerably, an issue that has widely been discussed with regard to reproducibility problems of study results (60, 61). In essence, to alleviate the influence of a single experimenter, systematically including several experimenters in one study, as we did here, is regarded a promising first step (62, 63).

## Conclusion

In the present study, we conducted two CJB paradigms and assessed their potential to detect differences in CJB induced via an affect manipulation. No differences in CJB were detected in either of the two tests, and no differences were observed in the EPM. Thus, all test outcomes point into the same direction. Given that the affect manipulation used here did not lead to the expected effects, the question whether the type of CJB paradigm used plays a role for the outcome remains unresolved. Since CJB paradigms often differ considerably, it would be crucial to further disentangle the potential impact of methodological details on test outcomes. This may be achieved by applying more effective affect manipulations. Furthermore, factors like reward sensitivity or the influence of food restriction could be addressed. Eventually, future studies should not only focus on mice, but be extended to other species.

## Data Availability

The datasets presented in this study can be found in the Figshare repository: https://figshare.com/s/1d613e1ba426d18f68c3.

## Supplementary material

The Supplementary material for this article can befound online at: https://www.frontiersin.org/articles/10.3389/fvets.2026.1813283/full#supplementary-material
