# Supplementary material for: Effects of enrichment removal on cognitive judgement bias of mice: a comparison of two paradigms
Source: Front Vet Sci. 2026 Apr 30;13:1813283. doi: 10.3389/fvets.2026.1813283 (PMC13171517; doi:10.3389/fvets.2026.1813283)
Supplement: Supplementary file 1 [file Supplementary_file_1.pdf]

Effects of enrichment removal on cognitive judgement bias of mice: A comparison of two paradigms

Viktoria Siewert[1,*], Louisa Bierbaum[1], Melanie Gleske[1], Carolin Mundinger[1], Rupert Palme[2], Sylvia Kaiser[1], Norbert Sachser[1], S. Helene Richter[1]

[1]Department of Behavioural Biology, University of Münster, Münster, Germany

[2]Department of Biomedical Science, University of Veterinary Medicine, Vienna, Austria

*correspondence: viktoria.siewert@uni-muenster.de

Supplementary Material

Table S1: Discrimination training steps of the TS paradigm. Discrimination training was conducted subsequent to a pre-training phase (for details see [1]) and consisted of six steps (modified from [2]). All sessions ended after maximally 30 min, unless the mouse reached the maximum number of trials before this time. During correction trials (CTs) animals were presented with the same cue until touching correctly. Pseudo-probe trials (balanced numbers of positive and negative trials that remained unpunished and/or unrewarded) were included to accustom the mice to the outcome of the probe trials during testing. Abbreviations: CPF: Cue presentation field, TF: Touch field, CT: correction trial.

Step	Max. number of trials	Description	Learning criterion	Return criterion	Correction trials (CTs)	Number of pseudo-probe trials
1	50	Top or bottom bar was presented in CPF. Crosses appeared on TFs with 2 s-delay. Correct TF had to be touched. Touches on incorrect TF did not result in any response.	Min. 5 days in this step. 50 trials in 20 min on 2 consecutive days. (In case of first re-entrance: 2 sessions sufficient for proceeding)	50 trials in 20 min not reached in 25 days → Pre-training	-	-
2	20 (+10)	Initially, 10 trials as in Step 1 were presented. Thereafter, both TFs could be touched after cue presentations. Correct touches were followed by a new trial. Incorrect touches were followed by a CT, until the mouse chose correctly.	80% correct responses and ≤7 CTs on two consecutive days	>20 CTs (except on first day in this step) or no CT reduction of 45% daily → Step 1	yes	-
3	50 (+4*)	Initially, 4 trials as in Step 1 were presented (*). Thereafter, training proceeded as in Step 2.	80% correct responses and ≤13 CTs on two consecutive days	>30 CTs or no CT reduction of 45% daily → Step 1	yes	-
4	50 (+4*)	Initially, 4 trials as in Step 1 were presented (*). Thereafter, training proceeded as in Step 2, but with a restriction of CTs to the initial 25/15/5 trials and the introduction of pseudo-probe trials afterwards.	80% correct responses and ≤8 CTs on two consecutive days	Learning criterion not met on 1 out of 4 days → Step 3	yes (in trials 1-25)	2 (pseudo-randomly distributed across trials 26-50)
5	50 (+4*)		80% correct responses and ≤6 CTs on two consecutive days	Learning criterion not met on 1 out of 4 days → Step 3	yes (in trials 1-15)	4 (pseudo-randomly distributed across trials 16-50)
6	50 (+4*)		80% correct responses and ≤4 CTs on two consecutive days	Learning criterion not met on 1 out of 4 days → Step 3	yes (in trials 1-5)	6 (pseudo-randomly distributed across trials 6-50)

Table S2: Discrimination training steps of the TUN paradigm. Discrimination training was conducted subsequent to a task-specific habituation phase, consisting of 4 days during which mice were accustomed to the apparatus and the rewards. The discrimination training comprised three steps (modified from [1]). All sessions ended after maximally 30 min, unless the mouse reached the maximum number of trials before this time. During correction trials (CTs) animals were presented with the same cue until touching correctly in the box where the initial error was made. Pseudo-probe trials were not included, because mice were rewarded during the actual probe trials in the test.

Step	Max. number of trials	Description	Learning criterion	Return criterion	Correction trials (CTs)
1	30	Long or short tunnel was presented. Only the correct reward hole was opened and mice had to collect the reward.	Min. 5 days in this step. 30 trials on 2 consecutive days.	Learning criterion not met after 5 additional sessions → Exclusion	-
2	30 (+4*)	Initially, 4 trials as in Step 1 were presented (*). Thereafter, both reward holes were opened. Correct choices were followed by a new trial. Incorrect choices were followed by a CT, until the mouse chose correctly.	80% correct responses on two consecutive days	-	yes
3	30 (+4*)	Initially, 4 trials as in Step 1 were presented (*). Thereafter, training commenced as in Step 2 but without CTs.	80% correct responses on two consecutive days	< 50 % correct responses to either of the two cues → Step 2	no

Table S3: Exploration of models for finding best random effects structure: Cognitive Judgement Bias Test Data. Bold: Model chosen for analysis. Abbreviations: AIC: Akaike information criterion; TS: Touchscreen, TUN: Tunnel.

Model	$AIC_{TS\ task}$	$AIC_{TUN\ task}$
*Choice ~ Affect manipulation * Cue + Experimenter + (1|ID) + (1|Age) + (1|Batch), family = binomial)*	483.85	441.38
*Choice ~ Affect manipulation * Cue + Experimenter + (1|Age) + (1|Batch), family = binomial)*	488.54	473.2
*Choice ~ Affect manipulation * Cue + Experimenter + (1|ID) + (1|Batch), family = binomial)*	481.85	439.4
*Choice ~ Affect manipulation * Cue + Experimenter + (1|ID) + (1|Age), family = binomial)*	481.85	439.38
*Choice ~ Affect manipulation * Cue + Experimenter + (1|age), family = binomial)*	489.48	474.91
*Choice ~ Affect manipulation * Cue + Experimenter + (1|Batch), family = binomial)*	489.92	484.52
Choice ~ Affect manipulation * Cue + Experimenter + (1|ID), family = binomial)	**479.85**	**437.4**

Table S4: Exploration of models for finding best random effects structure: Elevated Plus Maze Test Data. Bold: Model chosen for analysis. Abbreviations: AIC: Akaike information criterion; TS: Touchscreen, TUN: Tunnel.

Model	$AIC_{Distance}$	$AIC_{Open\ arm\ time}$	$AIC_{Open\ arm\ entries}$
*Behaviour ~ Affect manipulation * Group + Experimenter + (1|Age) + (1|Batch) + (1|Cage)*	401,59	-72,23	-100,88
Behaviour ~ Affect manipulation * Group + Experimenter + (1|Cage)	**398,92**	**-76,20**	**-104,81**
*Behaviour ~ Affect manipulation * Group + Experimenter + (1|Batch)*	407,23	-73,48	-103,87
*Behaviour ~ Affect manipulation * Group + Experimenter + (1|Age)*	410,97	-73,17	-103,63
*Behaviour ~ Affect manipulation * Group + Experimenter + (1|Batch) + (1|Cage)*	399,59	-74,23	-102,88
*Behaviour ~ Affect manipulation * Group + Experimenter + (1|Age) + (1|Cage)*	400,92	-74,20	-102,81
*Behaviour ~ Affect manipulation * Group + Experimenter + (1|Age) + (1|Batch)*	409,23	-71,48	-101,87

Table S5: Pairwise post-hoc comparisons of responses to the adjoining cues in both CJB tests. The statistical analysis was conducted using the “emmeans” package (version 1.11.0; adjust = "tukey"; [3]). TS: Touchscreen, TUN: Tunnel, P: Positive cue, NP: Near positive cue, M: Middle cue, NN: Near negative cue, N: Negative cue.

Task	Comparison	Z ratio	P value
TS	NP-P	-5.938	< 0.0001
	M-NP	-10.227	< 0.0001
	M-NN	5.995	< 0.0001
	N-NN	-0.768	0.94
TUN	NP-P	-4.338	0.0001
	M-NP	-3.936	0.0008
	M-NN	3.810	0.0013
	N-NN	-4.037	0.0005

Table S6: Statistical analysis of the Elevated plus maze test. Linear mixed models were fitted with the following fixed effects: Affect manipulation: 2 levels (enrichment removal, control), Group: 3 levels (TS = touchscreen, TUN = tunnel, HAND = handling), Experimenter: 2 levels (experimenter 1, experimenter 2); df: degrees of freedom.

**Model**	**Behaviour ~ affect manipulation * group + experimenter + (1|cage)**			
**Behaviour**	**Factor**	**χ^2**	**df**	**P value**
Open arm time (rel.)	Affect manipulation	0.0002	1	0.99
	Group	21.89	2	< 0.001
	Experimenter	6.94	1	< 0.01
	Affect manipulation : Group	1.62	2	0.45
Open arm entries (rel.)	Affect manipulation	< 0.001	1	0.98
	Group	20.19	2	< 0.001
	Experimenter	7.63	1	< 0.01
	Affect manipulation : Group	0.67	2	0.71
Total distance travelled (m)	Affect manipulation	0.59	1	0.44
	Group	2.17	2	0.34
	Experimenter	1.06	1	0.30
	Affect manipulation : Group	0.51	2	0.78

References

1. Krakenberg, V. *et al.* Technology or ecology? New tools to assess cognitive judgement bias in mice. *Behav. Brain Res.* **362**, 279–287. https://doi.org/10.1016/j.bbr.2019.01.021 (2019).
2. Bračić, M. *et al.* Once an optimist, always an optimist? Studying cognitive judgment bias in mice. *Behav. Ecol.* **33**, 775–788. https://doi.org/10.1093/beheco/arac040 (2022).
3. Lenth, R. *emmeans: Estimated Marginal Means, aka Least-Squares Means.* R package version 1.11.0 (2025). Available at: https://CRAN.R-project.org/package=emmeans
